# Vegetable Diet in the Chronic Diarrhœa of California

**Published:** 1852-06

**Authors:** S. Rush Haven

**Affiliations:** Chicago


ART. III.—*Vegetable Diet in the Chronic Diarrhœa of California.* By S. Rush Haven, M.D., of Chicago.

At the solicitation of a friend, I am induced to submit for the consideration of the readers of the *Journal*, a few ideas relative to the treatment of certain diseases as they appeared, and were observed by myself, during the autumn and summer of 1850—51 in the mountains of Upper California. It would perhaps be a work of supererogation to say to the scientific man that the diseases peculiarly incident to most mountainous regions, and particularly so of California, are mainly those of *deficient* or *depraved nutrition* and exposure to vicissitudes of climate.

The torrent of emigration that filled every gulch, ravine, and valley of the high mountain ranges of the upper country during the seasons referred to, when no other provisions could be obtained than salt pork and sea biscuit, could not, and did not, but result in developing an alarming amount of scorbutus and diarrhœa in all their protean forms and conditions.

Medical practice, unlike the laws of the Medes and Persians, is subject to change. This the experience of the last few years has amply demonstrated. In attempting to treat diseased conditions, having their origin in causes entirely remote and foreign from those of which we have received our impressions of similar diseases and their treatment, we too often find our science at fault and entirely inadequate to accomplish the end desired. The treatment of these maladies—especially the latter—according to the authorized practice in similar cases, was found, in the majority of instances, to result only in an aggravation of all the pathological conditions.

When the "books and schools" are "weighed in the balances and found wanting," the practitioner is justified in having recourse to the dictates of *common sense.*

A recourse to that in this instance suggested the propriety of supplying, or attempting to supply, in as direct and concentrated a form as possible, those materials for which the organism was languishing. This was evidently an exclusively vegetable diet.

Cases of chronic Diarrhœa, of that peculiarly obstinate type that is so seldom relieved by the ordinary modes of practice, were found to yield in an astonishingly short space of time upon the free use of grated raw potatoes and vinegar, without any (other) medication whatever. This kind of practice was found to be successful in too many cases not to be worthy of consideration. It will be noticed that no originality is claimed for the treatment of scorbutus by a vegetable diet, but that this diarrhœa or dysentery had none of the characteristics of scurvy, other than that of its occurring simultaneously under similar exciting causes. The rationale of this treatment is too evident for comment. How soon and how far it may obtain—viz.: building up instead of breaking down the tissues—in the treatment of many other diseases than those referred to in this paper, is a question of much import to the patient and practitioners.

